# Patterns of change in cardiovascular risk assessments and ankle brachial index among Puerto Rican adults

**DOI:** 10.1371/journal.pone.0245236

**Published:** 2021-01-20

**Authors:** Sabrina E. Noel, David J. Cornell, Xiyuan Zhang, Julia C. Mirochnick, Josiemer Mattei, Luis M. Falcón, Katherine L. Tucker

**Affiliations:** 1 Department of Biomedical and Nutritional Sciences, University of Massachusetts Lowell, Lowell, Massachusetts, United States of America; 2 Health Assessment Laboratory, University of Massachusetts Lowell, Lowell, Massachusetts, United States of America; 3 Center for Population Health, Zuckerberg College of Health Sciences, University of Massachusetts Lowell, Lowell, Massachusetts, United States of America; 4 Department of Physical Therapy and Kinesiology, University of Massachusetts Lowell, Lowell, Massachusetts, United States of America; 5 Department of Public Health, University of Massachusetts Lowell, Lowell, Massachusetts, United States of America; 6 Department of Nutrition, Harvard T.H. Chan School of Public Health, Boston, Massachusetts, United States of America; 7 College of Fine Arts, Humanities and Social Sciences, University of Massachusetts Lowell, Lowell, Massachusetts, United States of America; International University of Health and Welfare, School of Medicine, JAPAN

## Abstract

**Background:**

Puerto Rican adults have higher odds of peripheral artery disease (PAD) compared with Mexican Americans. Limited studies have examined relationships between clinical risk assessment scores and ABI measures in this population.

**Methods:**

Using 2004–2015 data from the Boston Puerto Rican Health Study (BPRHS) (*n* = 370–583), cross-sectional, 5-y change, and patterns of change in Framingham Risk Score (FRS) and allostatic load (AL) with ankle brachial index (ABI) at 5-y follow-up were assessed among Puerto Rican adults (45–75 y). FRS and AL were calculated at baseline, 2-y and 5-y follow-up. Multivariable linear regression models were used to examine cross-sectional and 5-y changes in FRS and AL with ABI at 5-y. Latent growth mixture modeling identified trajectories of FRS and AL over 5-y, and multivariable linear regression models were used to test associations between trajectory groups at 5-y.

**Results:**

Greater FRS at 5-y and increases in FRS from baseline were associated with lower ABI at 5-y (β = -0.149, *P* = 0.010; β = -0.171, *P* = 0.038, respectively). AL was not associated with ABI in cross-sectional or change analyses. Participants in low-ascending (vs. no change) FRS trajectory, and participants in moderate-ascending (vs. low-ascending) AL trajectory, had lower 5-y ABI (β = -0.025, *P* = 0.044; β = -0.016, *P* = 0.023, respectively).

**Conclusions:**

FRS was a better overall predictor of ABI, compared with AL. Puerto Rican adults, an understudied population with higher FRS over 5 years, may benefit from intensive risk factor modification to reduce risk of PAD. Additional research examining relationships between FRS and AL and development of PAD is warranted.

## Introduction

Puerto Ricans, the second largest Hispanic ethnic group on the US mainland [1], experience significant disparities in cardiovascular disease (CVD) risk factors compared with non-Hispanic whites and with Hispanics of other backgrounds [2]. Peripheral arterial disease (PAD) is associated with three times greater mortality [3], four times greater risk of myocardial infarction, and two to three times greater risk of stroke [4], and is associated with functional decline [5]. Although Hispanics overall have shown lower prevalence of PAD compared with non-Hispanic whites [6], among different Hispanic origins, Puerto Ricans have twice the burden of Mexican Americans [7]. For example, a recent study demonstrated that 42% of patients presenting to an outpatient clinic in Puerto Rico had PAD, providing additional evidence that this population may be at increased risk [8,9]. PAD is often asymptomatic [10,11], therefore, identification of early risk factors of PAD may lead to the development of prevention strategies to improve health outcomes in those at risk.

The Framingham Risk Score (FRS) is a commonly utilized measure that includes traditional CVD risk factors shown to predict 10-y risk of coronary heart disease (CHD) [12,13] and CHD-related events (e.g., stroke, myocardial infarction) [14]. Studies examining the relationship between FRS and PAD are scarce, including among Puerto Rican adults. In addition, as the FRS was developed and validated in a primarily non-Hispanic white population [15,16], it may inadequately determine CVD risk among Hispanic and other ethnic minority populations [16–19]. In particular, there may be a discordance between FRS classification and atherosclerotic indicators among Mexican Americans [19], due to high prevalence of risk factors within this population (e.g., waist circumference, CRP, etc.) which are not considered within the FRS criteria. Further, risk factors for developing PAD may differ between ethnic populations [6]. Therefore, further investigation of other potential predictors of atherosclerotic outcomes, including PAD, is warranted in this population.

Allostatic load (AL) reflects changes in the body’s regulatory systems resulting from sustained stress, leading to the development of chronic health conditions [20], including CVD [21,22] and PAD [23]. In contrast to risk factors included in the FRS, AL provides an aggregate measure of systemic physiological dysregulation occurring within an individual that ultimately leads to negative health outcomes [24] and extends beyond the traditional risk factors included in the FRS. Specifically, AL is a composite index of parameters including stress hormones and pro-inflammatory cytokines, as primary mediators of a stress response, as well as blood pressure, waist circumference, glycosylated hemoglobin, and cholesterol concentrations, as measures of cardiometabolic and immune dysregulation. Given the unique social, cultural, and environmental structures that contribute to chronic stress among Puerto Ricans [25,26], it is unsurprising that this population experiences higher AL than other populations [22]. Studies examining the relationship between AL and PAD have been cross-sectional, and conducted in non-Hispanic populations [23]. To our knowledge, the association between AL and ABI among Puerto Rican adults has yet to be examined.

Therefore, the purpose of this study was to examine the relationships between AL and FRS and 5-y follow-up ABI outcomes using cross-sectional, 5-y change, and patterns of change analyses among Puerto Rican adults from the Boston Puerto Rican Health Study (BPRHS). We hypothesized that both cross-sectional and changes in FRS and AL measures would predict ABI at 5-y follow-up.

## Material and methods

### Study population

**Fig 1:**
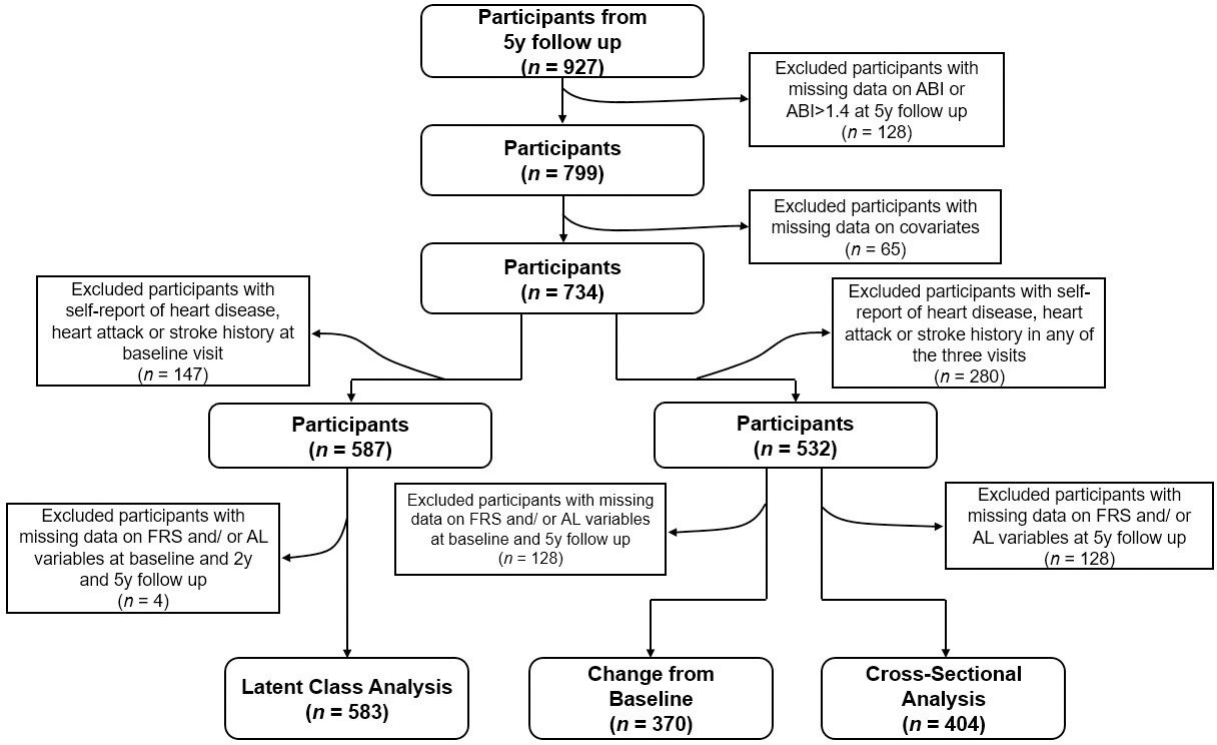
Flow chart of participation from the Boston Puerto Rican Health Study.

Data collected from 2004–2015 in the BPRHS, a longitudinal cohort aimed at determining the biological, social, lifestyle and environmental risk factors associated with health disparities experienced by Puerto Rican adults, were used in this analysis [26]. Briefly, 1499 Puerto Rican adults, aged 45 to 75 y, residing in the Boston area and self-identified as being of Puerto Rican origin, were recruited using data from high density 2000 census blocks and community approaches. Those with a Mini-Mental State Examination score ≤10, plans to move from the area within 2 y, or who were unable to answer questions due to a serious health condition were excluded. A total of 1267 participants completed a 2-y interview and 927 a 5-y interview. A total of 128 participants with missing ABI data at 5-y and 65 with missing information for covariates were excluded (Fig 1). In addition, 280 participants who self-reported having heart disease, heart attack or stroke at any of the three visits, were excluded from cross-sectional and longitudinal change analyses (*n* = 532), and 147 participants who self-reported having heart disease, heart attack or stroke at baseline were excluded from latent class analyses (*n* = 587). Participants with missing data on the FRS or AL at baseline, 2-y and 5-y were excluded from latent class analyses (*n* = 4), from baseline or 5-y follow-up for change analyses (*n* = 162) or at 5-y follow-up for cross-sectional analyses (*n* = 128). Therefore, 404 participants were available for cross-sectional analyses, 370 for 5-y change, and 583 for latent class analysis. All participants provided written informed consent. The Institutional Review Boards at the University of Massachusetts Lowell, Tufts University and Northeastern University approved all study protocols.

### Data collection and measurements

At all three time points, participants were interviewed in their homes by trained bilingual interviewers to collect sociodemographic information, health status, health behaviors and medication use. Anthropometric (height, weight, waist circumference) measurements were obtained in duplicate and averaged. Blood pressure was obtained at three time points during the interview using an electronic sphygmomanometer (Model HEM-71, Omron Healthcare) and an average of the last two measures was used. A 12-hour fasting blood sample was collected by a certified bilingual phlebotomist and a 12-hour urine sample was provided the day after or as soon as possible after the interview. Blood samples were analyzed for plasma high-density lipoprotein cholesterol (HDL-C) and total cholesterol, high-sensitivity C-reactive protein (CRP), glycosylated hemoglobin (HbA1c) and dehydroepiandrosterone sulfate (DHEA-S). An Olympus AU400 with enzymatic endpoint reaction was used to determine cholesterol concentrations (intra- and inter-assay CV% total plasma and HDL cholesterol concentrations were 1.8% and 2.2% and 3.0% and 7.0%, respectively). LDL-C was determined using the Friedewald formula for those values with total cholesterol < 4.52 mmol/L [27]. Serum glucose concentration was assessed by enzymatic kinetic reaction on the Olympus AU400 (intra and inter-assay CV% for serum glucose and insulin were 2.0% and 3.4%). Epinephrine, norepinephrine and cortisol concentrations were measured from urine samples. Fasting blood samples and ABI measurements were collected in participants’ homes by a trained and certified study phlebotomist.

### Primary exposures

#### Framingham risk score

Methodology by Wilson et al. [13] was used to calculate estimated 10-yr risk (%) of CVD. Risk factors included sex, age, diabetes, smoking, and predefined cut points for systolic and diastolic blood pressure, and total, LDL, and HDL cholesterol.

#### Allostatic load

A composite measure of AL (0 to 11 points) was calculated from 11 parameters representing five biological systems, including the hypothalamus-pituitary-adrenal (HPA) axis and the cardiovascular/metabolic, neuroendocrine, inflammatory and sympathetic nervous systems. The sum of the following parameters, for which participants fell into the upper or lower clinical cut points or established quartiles (serum DHEA-S, epinephrine, norepinephrine and cortisol), was used: HDL-C (<40 mg/dl), total cholesterol (>240 mg/dl), systolic blood pressure (>140 mm Hg), diastolic blood pressure (>90 mm Hg), HbA1c (>7%), waist circumference (>102 cm for men or >88 cm for women), DHEA-S (≤589.5 ng/ml for men or ≤368.5 ng/ml for women), cortisol (>41.5 μg/g creatine for men or >49.5 μg/g creatine for women), epinephrine (>2.8 μg/g creatine for men or >3.6 μg/g creatine for women), norepinephrine (>30.5 μg/g creatine for men or >46.9 μg/g creatine for women), and CRP (>3 mg/L) [28–33]. Individuals received one point for values outside of each specified cut point. A point was also assigned to components in the normal range, only in the presence of medication use for diabetes, hypertension, hyperlipemia, or testosterone, as these individuals may have normal values that are artificial due to medication use.

### Outcome measure

#### Ankle-brachial index

Systolic blood pressure was measured in the right and left brachial arteries and posterior tibial arteries with an ultrasonic doppler (Nicolet Pocket-Dop II, Natus Medical Inc., Pleasanton, CA), following previously published methods [34]. Prior to data collection, participants rested quietly in a supine position for a minimum of 5 minutes. If pulse was absent from posterior tibial arteries, dorsalis pedis arteries were utilized instead. An average of two measures was used to calculate ABI for each side and the lower of the two values was utilized to define ABI for each participant. Participants were excluded from lower extremity ABI measures if they had a venous stasis ulceration, if occlusion pressure could not be reached, and/or if they were actively being treated for deep vein thrombosis, and from upper extremity ABI measures if they had undergone a mastectomy.

#### Covariate assessment

Sociodemographic variables were assessed at all three visits through questionnaire, including: age (y), sex (male/female), and educational attainment (<8^th^ grade; 8-12^th^ grade or GED; some college, bachelor’s, or graduate degree). Health behaviors, including smoking (never, past, current), alcohol consumption (none, moderate, heavy), and physical activity using a modified Paffenbarger questionnaire [35,36], were also collected at each visit. Lastly, white blood cell count (1000/uL) was determined on a HORIBA ABX Pentra 60C+ analyzer using whole blood.

#### Statistical analyses

All data were analyzed using SAS software (version 9.4, SAS Institute) and MPlus 7 [37]. Assumptions of normality of all continuous variables were examined via visual inspection of distribution histograms. Separate models were examined for FRS and AL. Cross-sectional associations between FRS and AL with ABI at 5-y follow-up were examined using multivariable linear regression models (*n* = 404). Multivariable linear regression was also used to model changes in FRS and AL from baseline to 5-y follow-up in relation to 5-y ABI, adjusting for respective baseline measures (*n* = 370). Latent growth mixture modeling (LGMM) was used to capture unobserved heterogeneity over time and identify different latent trajectories. LGMM models were fitted for two, three, and four latent groups, using longitudinal data (baseline, 2-y, and 5-y follow-up) from all available FRS and AL scores (*n* = 583). Each participant was assigned to one of the latent groups, which were determined using estimated intercept and slope of individuals with similar trajectory. Multivariable linear regression was then used to test associations between FRS and AL trajectory groups and ABI. Models including FRS were adjusted for education, alcohol consumption, and physical activity score. Models with AL were adjusted for sex, age, education, physical activity, alcohol consumption, smoking status, and white blood cell count (to account for potential current infection). We conducted analyses with ABI as a continuous variable, as only 53 participants had an ABI indicative of likely PAD (≤0.9) at 5-yr follow-up.

## Results

Participants were primarily female (73%) older adults with low educational attainment, and were physically inactive (Table 1). The majority did not consume alcohol (54.4%) and 48% never smoked. A total of 33.0% had diabetes and mean (95% CI) BMI was 31.8 (95% CI = 31.3, 32.3) kg/m^2^.

**Table 1:** Participant demographics at baseline (*n* = 583).

**Categorical Variables**	**Count**	**Percent**
Female	425	72.9
Education		
Less than 8^th^ grade	268	46
9^th^– 12^th^ grade	235	40.3
Some college, bachelor’s or graduate degree	80	13.7
Smoking		
Never	282	48.4
Past	162	27.8
Current	139	23.8
Alcohol		
None	313	54.1
Moderate	226	39
Heavy	40	6.9
Diabetes	190	33.1
Hypertension	369	63.8
**Continuous Variables**	**Mean ± SD**	**95% CI**
Age, yrs	55.9 ± 7.22	55.3, 56.5
Height, cm	159 ± 8.28	158, 159
Weight, kg	79.8 ± 17	78.4, 81.2
Body mass index, kg/m^2^	31.8 ± 6.49	31.2, 32.3
Waist circumference, cm	0.7 ± 0.46	0.66, 0.74
Physical activity score	31.8 ± 4.59	31.4, 32.2
Systolic blood pressure, mmHg	134 ± 18.8	132, 135
Diastolic blood pressure, mmHg	81.6 ± 10.5	80.7, 82.4
HDL cholesterol, mg/dl	45.4 ± 12.5	44.4, 46.4
LDL cholesterol, mg/dl	111 ± 33.3	109, 114
Total cholesterol, mg/dl	187 ± 38.8	184, 191
Urinary cortisol, μg/g	33.8 ± 30.9	31.3, 36.4
Urinary epinephrine, μg/g	3.73 ± 3.42	3.45, 4.02
Urinary norepinephrine, μg/g	37.6 ± 27.5	35.3, 39.8
HbA1c, %	6.8 ± 1.64	6.66, 6.93
DHEA-S, ng/ml	884 ± 652	830, 937
C-reactive protein, mg/l	6.2 ± 8.9	5.47, 6.93
White blood cell count, 1000/μl	6.77 ± 1.86	6.62, 6.93
Framingham Risk Score, %	11.3 ± 7.26	10.7, 11.9
Allostatic Load	4.2 ± 1.85	4.05, 4.36
Ankle-brachial index (5-y follow-up)	0.99 ± 0.08	0.98, 0.99

Note: Values are percentages for categorical variables and means ± standard deviation (SD) and 95% confidence intervals (95% CI) for continuous variables.

DHEA-S, Dehydroepiandrosterone-sulfate; HbA1c, hemoglobin A1c test; HDL, high-density lipoprotein; LDL, low-density lipoprotein.

In cross-sectional analyses, greater FRS was associated with significantly lower ABI at 5-y (β = -0.149, 95% CI = -0.262, -0.037, *p* = 0.010) after adjusting for covariates (Table 2). AL at 5-y, however, was not associated with ABI at 5-y (β = -0.003, 95% CI = -0.007, 0.002, *p* = 0.252) after adjusting for covariates. Change in FRS from baseline to 5-y was associated with lower ABI at 5-y (β = -0.171, 95% CI = -0.332, -0.009, *P* = 0.038), after adjusting for covariates and baseline FRS. Change in AL was not associated with ABI at 5-y, after adjustment (β = -0.001, 95% CI = -0.007, 0.004, *P* = 0.63). The mean (95% CI) for FRS and AL were: 10.6% (10%, 11%) and 4.1 (3.95, 4.33) at baseline; 11.0% (10%, 12%) and 4.3 (4.08, 4.46) at 2-y follow-up; and 11.4% (11%, 12%) and 4.8 (4.59, 4.96) at 5-y follow-up, respectively.

**Table 2:** Cross-sectional, 5-y change, and latent class analyses for the relationship between FRS, AL, and ABI.

		ABI at 5-y Follow-Up			
		β		95% Confidence Interval	*P*
**Cross-Sectional at 5 y (*n* = 404)**					
FRS^a^		-0.149 ± 0.057		-0.262, -0.037	*0.010
AL^b^		-0.003 ± 0.002		-0.007, 0.002	0.252
**5-y Change from Baseline (*n* = 370)**					
Change in FRS^a^		-0.171 ± 0.082		-0.332, -0.009	*0.038
Change in AL^b^		-0.001 ± 0.003		-0.007, 0.004	0.629
**Latent Class Analyses (*n* = 583)**					
FRS^a^	Low-Ascending (*n* = 51)		-0.025 ± 0.012	-0.049, -0.001	*0.044
	No Change (*n* = 532)		Ref		
AL^b^	Low-Ascending (*n* = 296)		-0.016 ± 0.007	-0.030, -0.002	*0.023
	Moderate-Ascending (*n* = 287)		Ref		

Note: Boldface indicates statistical significance (*P < 0.05).

^a^ Models with FRS were adjusted for educational attainment, physical activity score, and alcohol consumption.

^b^ Models with AL were adjusted for sex, age, educational attainment, physical activity score, alcohol consumption, smoking and white blood cell concentration.

ABI, ankle brachial index; AL, allostatic load; FRS, Framingham risk score.

**Fig 2:**
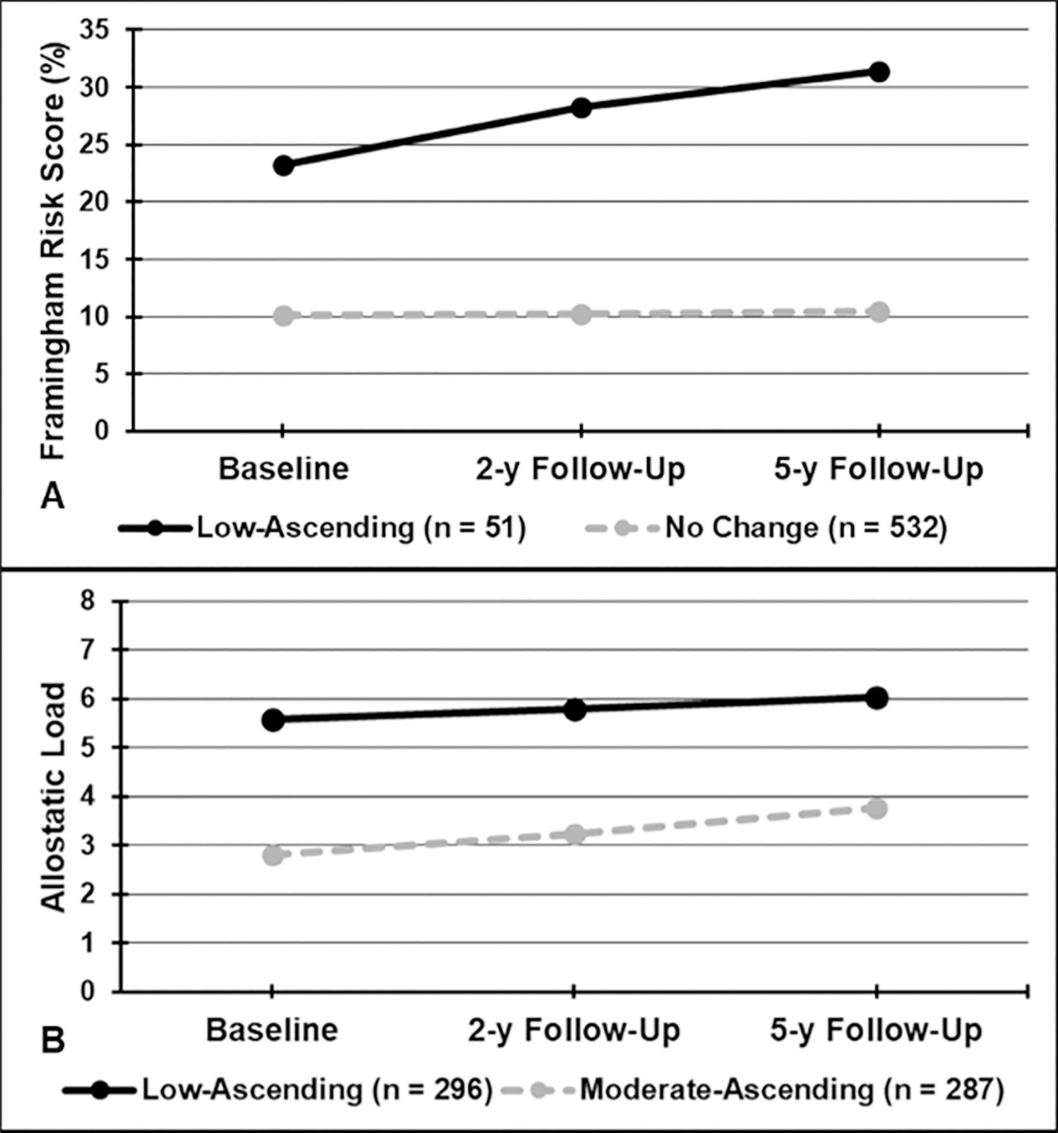
Predicted Trajectories for: A) Framingham Risk Scores; and B) Allostatic Load.

Based on the fit indices for the estimated latent growth mixture models, two-model solutions were selected for FRS and AL, and these categorical variables were used in the analyses. Fig 2A demonstrates the two trajectories for FRS: 1) low-ascending FRS; and 2) no change in FRS; and Fig 2B demonstrates the two trajectories for AL: 1) low-ascending AL; and 2) moderate-ascending AL. In multivariable analysis, participants in the low-ascending FRS had significantly lower ABI, compared with those who were had no change in FRS (β = -0.025, 95% CI = -0.049, -0.001, *P* = 0.044) (Table 2). Participants in the moderate-ascending AL trajectory had significantly lower ABI, compared with those in the low-ascending AL trajectory, after adjusting for covariates (β = -0.016, 95% CI = -0.030, -0.002, *P* = 0.023).

## Discussion

In this study, greater FRS at 5-y, change in FRS (baseline to 5-y), and trajectory of increasing FRS score over 5 y, were associated with lower ABI at 5-y follow-up among Puerto Rican adults. While we hypothesized that both FRS and AL would be significantly associated with ABI, our findings suggest that only a trajectory of moderate-ascending AL scores was associated with ABI at 5-y, compared with a trajectory of low-ascending AL. This work highlights the utility of the FRS in identifying Puerto Rican older adults at risk for cardiovascular events, a population that is relatively understudied and underrepresented in cardiovascular research. These findings are novel, as few studies have examined the association between FRS and ABI outcomes among Hispanics [38], and none within the Puerto Rican population. In addition, to date, only one previous study has examined the relationship between AL and ABI, also cross-sectional in nature [23].

PAD can result in claudication pain, ulcerations, and limb amputation, and is a significant predictor of other cardiovascular outcomes [3,4]. Although an ABI ≤0.9 is a clinical screening measure of PAD [39], many patients are asymptomatic, and therefore do not present with symptoms indicating ABI assessment, subsequently resulting in under diagnosis [40]. As such, it is not surprising that the presence of PAD remains undetected and untreated by primary care physicians in 70% of asymptomatic individuals [41]. However, these asymptomatic individuals may benefit most from interventions aimed at modifying major risk factors for PAD.

Risk factors for PAD, such as smoking, poor dietary quality, hypertension, hyperlipidemia, diabetes, and obesity, are similar to those for CVD [42–44]. Clinical management of PAD focuses on reducing symptoms and morbidity and mortality through modification of these known risk factors [45]. Although it had been previously proposed to incorporate ABI in the FRS to improve prediction of cardiovascular risk [46], a 2013 systematic review for the US Preventative Services Task Force concluded that the addition of ABI to FRS had limited value in predicting CHD and CVD [47]. The FRS was also developed using data predominantly from non-Hispanic white adults [13,48] and has been shown to systematically overestimate 5-y risk of CHD for Hispanic men [14]. However, these results were based on data from an older cohort study in Puerto Rico (1965–1968) and, to our knowledge, no study has assessed the FRS among Puerto Ricans living on the U.S. mainland.

Our findings indicate that higher FRS (cross-sectional at 5-y and change from baseline to 5-y) is associated with lower ABI at 5-y follow-up among Puerto Rican adults aged 45 to 75 y. Further, individuals in the trajectory class with low-ascending FRS compared with individuals in the class with no change in FRS had lower ABI at 5-y follow-up, suggesting that this group may benefit from strategies to manage and reduce FRS risk factors. Given the fact that PAD is often asymptomatic [10,11], the FRS may serve as a screening tool to identify Puerto Rican adults at risk of developing PAD and, therefore, should be referred to risk factor modification programming by healthcare providers. This is of public health importance for this high-risk and underserved population, as Puerto Rican adults have significant disparities in many FRS risk factors, including low HDL-cholesterol [49], high blood pressure, and obesity [26].

One previous study [23], found that an AL score >4 was associated with higher odds of PAD after adjusting for race/ethnicity, age and sex (OR = 2.0) [23]. AL represents the dysregulation of interrelated physiological systems resulting from prolonged activation of the autonomic nervous and HPA systems in response to sustained stressors and other psychosocial factors, and is associated with health outcomes, such as CVD [50–52]. Given that Puerto Rican adults experience higher AL at younger ages, compared with other populations [22,53], and that increased AL is a stronger predictor of abdominal obesity and hypertension than presence of metabolic syndrome in this population, we hypothesized that AL may be a stronger predictor of ABI outcomes compared with more traditional risk assessment methods [22]. In contrast to this hypothesis, the current study did not demonstrate an association between ABI at 5-y and AL as a continuous variable. This finding was surprising given the fact that AL utilizes a more expansive set of variables to derive the overall AL score than the FRS. However, a moderate-(vs. low-) ascending AL trajectory from baseline to 5-y was significantly associated with lower ABI at 5-y. This suggests that AL may identify a sub-set of individuals at risk for PAD in those with increasing AL scores over 5-y. This is important, as a single AL score may not be clearly associated, but a trend of worsening AL scores may suggest increased risk of PAD and cardiovascular events. That said, for screening purposes AL would not be recommended and the clinical utilization of FRS is likely to be more cost-effective and feasible, compared to obtaining biomarkers necessary to quantify AL. It is also possible that the upper or lower clinical cut points for deriving the overall AL scores may mask underlying cardiovascular changes across time, which may limit the ability of AL to predict ABI outcomes.

This study had several strengths, including the use of data from one of the largest cohorts of Puerto Rican adults living on the U.S. mainland, with a comprehensive assessment of sociodemographic and health behavior information as well as the biological measures required to calculate FRS and AL. In addition, measures of FRS and AL were available at three time points, which enable the assessment of trajectories over 5-y in this population. However, this study is limited in that ABI measures were only available at 5-y follow-up. Furthermore, the limited number of participants with ABI ≤0.9 in the BPRHS sample (*n* = 53) did not allow for assessment of the likelihood of developing PAD, making comparison of PAD incidence to previous studies difficult. Additional limitations include reduced sample size (*n* = 370–583) after excluding participants with cardiovascular disease, missing data, and low percentage of males (27.3%). Therefore, future longitudinal studies are needed to examine associations between changes in FRS and AL with changes in ABI, as well as with incidence of PAD, particularly within high-risk populations.

## Conclusions

Our findings suggest that the FRS may be a better predictor of ABI outcome than AL among Puerto Rican adults. Accordingly, the FRS may serve as a feasible and cost-effective clinical risk assessment tool to identify individuals at risk for PAD who would benefit from interventions targeting modifiable risk factors associated with its progression. This may be particularly important for asymptomatic individuals and for high-risk and underserved populations. Further research is needed to examine these associations in other ethnic and minority populations, and with longitudinal ABI measures and risk of PAD.
